# Community Response to Noise from Hot-Spots at a Major Road in Quito (Ecuador) and Its Application for Identification and Ranking These Areas

**DOI:** 10.3390/ijerph19031115

**Published:** 2022-01-20

**Authors:** Virginia Puyana-Romero, Jose Luis Cueto, Giuseppe Ciaburro, Luis Bravo-Moncayo, Ricardo Hernandez-Molina

**Affiliations:** 1 Grupo de Investigación Entornos Acústicos, Departamento de Ingeniería en Sonido y Acústica, Campus Granados, Universidad de Las Américas, Quito 170125, Ecuador; luis.bravo@udla.edu.ec; 2 Laboratorio de Ingeniería Acústica, Campus de Puerto Real, Universidad de Cádiz, 11510 Puerto Real, Spain; joseluis.cueto@uca.es (J.L.C.); ricardo.hernandez@uca.es (R.H.-M.); 3 Dipartimento di Architettura e Disegno Industriale, Università degli Studi della Campania “Luigi Vanvitelli”, Borgo San Lorenzo, 81031 Aversa, Italy; giuseppe.ciaburro@unicampania.it

**Keywords:** dose–response indicators, noise hot-spot, major road traffic noise, noise action plans, noise survey, noise legislation

## Abstract

Environmental legislation in Ecuador is advancing with the legitimate aspiration of providing citizens with new standards of quality and environmental health. In the context of environmental noise, these legislative advances are based on the experience accumulated in other countries, which is an advantage that must be managed with caution by incorporating local factors into noise management procedures. This study advances two lines of work. The first is to survey the population about their attitude towards noise from a major road to try to detect local factors in the annoyance and sleep disturbances. The second uses this information to compare noise indicators for the detection and ranking of hot-spots from major roads. The interviewees exhibited a high level of annoyance and sleep disturbance due to noise compared with the results of other studies. Results show that there are small differences in the definition of hot-spots when using WHO’s dose–response curves for *L_den_* ≥ 68 dB for and for *L_night_* ≥ 58 dB, in comparison with the curves generated in this study (CS). Regarding the application of both dose–response curves (WHO vs. CS) to the estimation of the population at risk of the harmful effect of nighttime traffic noise (HSD), small oscillations are also observed even when *L_night_* ≥ 58 dB and *L_noche_* ≥ 60 dB are used.

## 1. Introduction

The noise maps fulfill the function of diagnosing environmental acoustic quality throughout the city, but the process will not be closed until the action plans against noise are carried out. It is important that these first action plans are perceived as effective by citizens, and therefore the correct identification of noise problems is essential for the suitable management of noise environmental health. In Ecuador, in the first round of noise mapping in an agglomeration of more than 250,000 inhabitants, only the main road network has to be considered [1] in the evaluation of traffic noise. Although there are limits (for *L_dia_* and *L_noche_*) for various types of noise sources, Ecuadorian legislation does not explicitly set a target for long-term dose levels (nor specifically due to road traffic) to preserve the acoustic quality of the different types of noise zoning.

Generally speaking, road traffic noise may not be the source of noise that raises the most complaints from citizens, but its extent makes it the main source of noise pollution, causing a significant impact on human health. In recent decades, various research studies have shown that exposure to environmental noise poses a significant risk to physical and mental health [2,3,4,5], generating a large burden of disease associated with the exposure dose [6]. The World Health Organization (WHO) [3,4] warns that the negative consequences for the population occur even below the levels recommended for evaluation on strategic noise maps by the Environmental Noise Directive (END) and proposes recommended exposure values for the protection of public health according to the type of source of noise. In particular, for traffic noise, the WHO recommends assessing the *L_den_* = 53 dB (*L_den_* is the END noise indicator that corresponds to the average noise level throughout the day, evening, and night) and the *L_night_* = 45 dB [3]. Although these are the recommendations set as a goal in the coming years by the WHO, in some developing countries, it will be necessary to live for a time with less high expectations, in line with provisional recommendations that are more accessible in the short term (for example *L_night_* = 55 dB [7]). In that regard, there was a consensus among members of the Guideline Development Group [3] not to provide interim targets, as they are not based on health evidence, so vulnerable groups cannot be protected using these criteria. However, different EU member states generate noise regulations with the most varied limit values/guidance values when considering noise action plans for major roads, with the highest values found being *L_den_* = 70 dB and *L_night_* = 60 dB [8]. These values will be considered as reference values for this study, setting the threshold of what is considered a high exposure to road traffic noise.

END established the necessity of evaluating the cumulative effects of noise on population health, employing dose–response relations [9,10,11,12] using *L_den_*, which was related to the annoyance measure, and *L_night_*, which was related to the sleep disturbance measure. In that regard, at the beginning of 2020, the END incorporated the WHO’s scientific findings (e.g., [3,13,14,15]) as a method of evaluation of the effects of road noise applicable to Europe. The strength of the EU in the generation of legislation and procedures can inspire other countries that lag behind to systematically incorporate these findings.

The idea behind the present study is to extract lessons that can be introduced in Ecuador. Orderly development of policies that provide adequate responses for the management of the health risks regarding noise requires a more in-depth approach to the analysis of local factors. Not surprisingly, the WHO [3] prevents the validity of direct transferability of noise-exposure–response relationships describing noise annoyance from locations where studies were carried out or where data were otherwise gathered to other locations. This heterogeneity of the study estimates is largely due to non-acoustic factors, such as cross-cultural differences in the experience that citizens observe about noise [3,16,17]. Some previous studies have searched for evidence for the deviation from the accepted models of noise-exposure–response relationships due to road traffic concerning annoyance and self-reported sleep disturbances [18,19,20,21,22,23]. On the other hand, the aforementioned WHO noise-exposure–response curves for roads are based on cross-sectional studies developed only in cities in Europe and Asia-Pacific. For these reasons, surveys are carried out on a sample of the population living in areas exposed to high levels of road traffic noise in a study area of Quito, using a questionnaire to ask about the annoyance and sleep disturbance. This is the main justification of the study that is presented here. Other aspects that will be discussed have to do with the applicability of the findings that can be extracted from the main line of work.

In this way, the results obtained from the survey will be used to clarify whether local factors can influence the characterization of noise hot-spots in this type of infrastructure and how. Noise hot-spots from road infrastructure are defined in this study as “spatial units” where people live, who, as a result of their exposure to traffic noise, are at the highest risk of suffering a significant adverse impact on their health and quality of life [24].

Ranking the noise problem becomes an easier task if the distribution of people exposed to different noise levels has been previously condensed into a single variable (a dose–response indicator or any other type of community noise assessment indicator based on scientific evidence). A single variable is easier to handle and facilitates the comparison of hot-spots and therefore can better assist authorities in decision-making. There is a lot of literature covering different indicator proposals [25,26,27,28,29,30,31,32].

### Objectives and Hypotheses

Taking into account the context in which the research is conducted, the proposed objectives are the following:

(i) Estimate the dose–response curves of annoyance and sleep disturbances extracted in a high-density traffic section of a major road in Quito.

(ii) Compare the resulting noise hot-spots with various noise indicators, including those generated by dose–response curves extracted in a high-density traffic section of a major road in Quito.

The following hypotheses were formulated in order to develop the objectives set for the study:

**Hypotheses** **1** **(H1).** *The dose–response curves for noise annoyance and sleep disturbance calculated for the study area are within the confidence intervals of the WHO dose–response curves.*

**Hypotheses** **2** **(H2).** *There will be no significant differences between the hot-spots calculated using the defined noise indicators.*

## 2. Material and Method

The methodology that follows is designed to respond in the most successful way to accomplish the objectives of the research. To do this, the methodology has been divided into 4 phases, identification of the case study, survey campaign, calculation of the dose–response curves, and comparison of indicators, whose justification will be addressed during their presentation.

### 2.1. Case study: Quito’s Bypass

**Figure 1:**
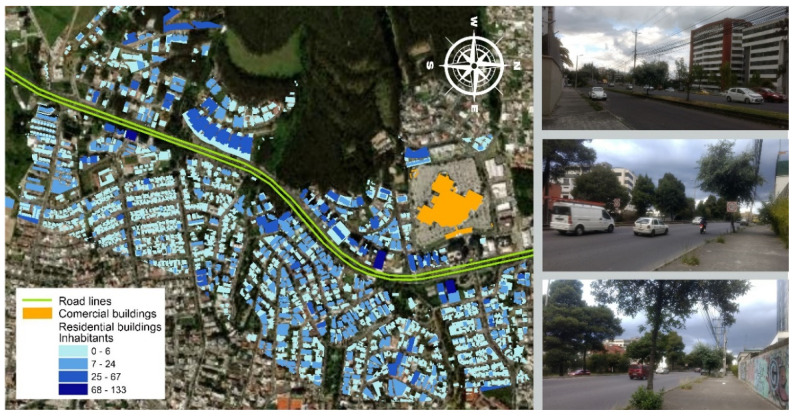
(**Left**): Study area. The total map comprises 12,592 inhabitants and approximately 3 Km of the highway. The study area considers the buffer that contains people exposed to the proposed lower limits of noise recommended by WHO. Only the stretch of 2640 m of affected people living in homes is analyzed, and it is shown on the map. (**Right**): Photographs of different sections of the highway.

Quito is located at 2800 m above sea level, encased in a narrow valley between mountains in which temperature inversions are a normal phenomenon. This is relevant for noise propagation and particle matter dispersion. According to the ranking prepared by INRIX, Quito was the eighth most congested city in the world in 2020, due to the geographically constrained environment and deficient quality and size of the infrastructure [33]. The main road network of Quito is structured by an urban peripheral ring, made up of Simón Bolívar Avenue to the east and Mariscal Sucre Avenue to the west. These highways play a fundamental role in the journeys of the city, since they connect different neighborhoods in the north–south axis and constitute a collector of local, provincial, and national flows. The study area is the stretch of Mariscal Sucre Avenue between Mañosca and Edmundo Carvajal Streets (Figure 1). This highway stretch is located on the slope of the Pichincha volcano, and due to the terrain, the buildings located on both sides of the highway are built on a slope or on platforms designed to level the ground. The capacity of the infrastructure is given by the three lanes per direction, and that is why the value of the AADT (annual averaged daily traffic) exceeds 100 thousand vehicles, an estimation consistent with other studies on the area [34]. The study area is predominantly residential, with multistorey building units, and has a very important mall in the city. These characteristics that combine high population density and high traffic flows have made this area attractive for the study. There is a reasonable knowledge of the noise behavior of Mariscal Sucre Avenue since traffic and noise measurement campaigns have been carried out on different days. The shapefiles used for the construction of the noise maps were from a 2014 database downloaded from the webpage of the Secretaría General de Planificación of Quito [35], including information on the density of people living in the area.

To obtain the data on the most exposed population—of which we conducted the survey—a façade noise map of the study area was calculated. This map allowed us to obtain knowledge of the spatial distribution of the traffic noise levels to which the population is exposed. The results of the noise mapping exercise can be consulted in the Supplementary Material File S1.

### 2.2. Survey Campaign

To evaluate the levels of exposure and the degree of annoyance and sleep disturbances due to noise in the study area, a socio-acoustic survey was carried out.

#### 2.2.1. Ethical Aspects and Data Protection

The ethical aspects of the research project (including the survey) were evaluated before the start of the project by the Committee of Ethics of Investigation in Human Beings (of the Office of Scientific Integrity) of the University of Las Americas (CEISH-UDLA), which determined that a specific approval was not necessary, as the research involves minimal risk for the participant and the collected data are de-identified (Document OIC-CEISH-UDLA-2021-06-15-001). Data management fulfills the data protection policy in force in Ecuador.

#### 2.2.2. Questionnaire Design

The questionnaire used for the survey campaign was designed following the recommendations of the ISO-15666 and the International Committee for the Biological Effects of Noise (ICBEN) Team, “Community Response to Noise” [36,37]. A pre-test was conducted to evaluate the validity and length of the questionnaire, in which 15 students and teachers of the university participated. In this pre-test, participants were asked if they would finish and deliver the questionnaire (if they were at their homes). According to the responses to that question, and to increase the participation, preserving the aim of the study, some questions were removed from the original version of the questionnaire.

The final questionnaire expressly indicated that the surveys had to be filled in individually and that all tenants living at the same address could complete the survey. It was a written questionnaire, distributed using the distribute–collect method. The survey campaign was conducted by the company of social studies Preinvespla in July–August 2021. For security reasons, the questionnaires were given to the building security guards, who distributed them to the tenants who agreed to participate.

A total of 251 questionnaires with socio-acoustic information were collected during the survey campaign, constituting a response rate of 60.2%. This percentage includes only the relationship between questionnaires distributed to tenants and collected. It does not include the inhabitants of those buildings in which the delivery of the questionnaires was not allowed or those inhabitants who did not accept the questionnaire. Participants were asked to write down their address in order to locate their homes in the façade noise map (and consequently identify the noise levels they were exposed to) and to indicate whether they saw Mariscal Sucre Avenue from their living room and their bedroom. The exclusion criteria were the non-location of the building in the study area, the exposure to levels *L_den_* < 53 dB and *L_night_* < 45 dB, and the lack of direct vision of the highway from both the living room and the bedroom. Following these criteria, 7 questionnaires were excluded from the statistical analysis.

The questionnaire was structured in three parts. The first part contained general questions about age, gender, address, number of people living in the home, and highway visibility. The questions of the second part were about noise annoyance, workplaces during the pandemic, degree of noise interference in the performance of work, noise complaints from other members of the family unit, and perception of the degree of annoyance before and during the pandemic. They were also asked about other noise sources that were more annoying than the noise coming from Mariscal Sucre Avenue, if they perceived noise from the avenue with open windows and with closed windows, and if the noise forced the window to be closed. The third part contained questions about sleep disturbances due to traffic noise from the study highway and about the degree of sleep disturbance before and during the pandemic. The questions referred to the period of one year. More information about the main questions of the first, second, and third parts of the questionnaire given to the participants can be found in the Supplementary Material File S2.

The 5-point Likert scale proposed by the ISO-15666 for the Spanish version was assessed in the pre-test. In one of the questions, the subjects were asked to choose the most appropriate adverb for each level of the scale. Four adverbs/expressions were used in each level, according to the Spanish version suggested by the ISO-15666 (1. Absolutamente nada, 2. Ligeramente, 3. Medianamente, 4. Mucho, and 5. Extremadamente) and three more chosen by Ecuadorian students and teachers of the university. The expressions selected by more participants of the pre-test were used. They coincide with the ones of the ISO-15666 at levels 1, 4, and 5 of the scale; levels 2 and 3 were substituted by “2. Un poco” and “3. Moderadamente”. Finally, the questions on noise annoyance and sleep disturbance included in the questionnaire were rated on a 5 points Likert scale (1. Not at all, 2. A little, 3. Moderately, 4. A lot, and 5. Extremely).

In this study, the variable %HA_CS (hereinafter CS will be the abbreviation for “case study”) is the percentage of “highly annoyed” study participants. The “highly Annoyed” (HA_CS) category includes participants who choose a high position on the annoyance response scale. Similarly, the variable %HSD_CS is the percentage of “highly sleep-disturbed” (HA_CS) participants. Following the standard proposed by the ICBEN [37], the upper two categories of the 5-point response scale were used for defining “highly annoyed” and “highly sleep-disturbed“ participants. The questions “How much has traffic noise from Mariscal Sucre Avenue annoyed or disturbed you at home?” and “How much did traffic noise from Mariscal Sucre Avenue alter your sleep at night?” were used for the calculations of %HA_CS and HSD_CS, respectively.

The noise levels *L_den_* and *L_night_* to which the home of each participant was exposed were obtained from the façade noise map according to the address written in the questionnaires. These noise levels and the survey data were linked through Geographic Information System using ArcGis Desktop 10.8.

#### 2.2.3. Data Analysis and Statistical Test for Treating the Results of the Surveys and for the Calculation of the Dose–Response Curves

Spearman’s correlations and Cohen’s standard were used to evaluate the association between variables. Quadratic and linear regression models were calculated to obtain the dose–response curves for the case study. The internal consistency of the data was assessed with Cronbach’s alpha. The software R was used for the data analysis. Further information on the test and software used for the data analysis can be found in the Supplementary Material File S4.

### 2.3. Comparison of the Hot-Spot of the Study Area Calculated with Different Community Noise Assessment Indicator

One of the objectives of the study is the comparison of the hot-spots calculated with the dose–response curves for the study area and the ones defined by the WHO [14,15]. The present research uses a 3D GIS-based tool for the identification and prioritization of the hot-spots on major roads (HSIP 3D-tool for short). An extensive description of the tool applied is discussed in the research article [38] and the Supplementary Material File S3.

Once an environmental noise limit value has been set for *L_den_*, all *L_den_*-based indicators (or *L_night_*-based) will describe the same extent of highway responsible for people’s exposure (they all define the same intervention area). However, the different noise indicators will give a different vision of the spatial distribution of the noise problem (hot-spots) and the urgent areas of intervention. The idea behind this proposal is to understand how the possible combinations of indicators point to different assessments scenarios. The *L_den_* and *L_night_* values from which the comparative analysis was performed were the lower limits from which the dose–response curve of the study carried out on Avenida Mariscal Sucre was defined. The study focused on populations highly exposed to noise; these values (as will be seen later) are *L_den_* ≥ 68 dB and *L_night_* ≥ 58 dB. The *L_noche_* level equivalent to *L_night_* was also used after performing a conversion. The community noise assessment indicators (6 in total) that were incorporated into the HSIP 3D-tool calculations to compare their results are the following:

(i) Pop. Population living above the noise limits considered for *L_den_*, *L_night_*.

(ii) HA and HSD. The total number of people (N) affected by the harmful effects of level of noise due to road noise exceeding the proposed limits for *L_den_* and *L_night_* and calculated with the proposed dose–response curves in annex iii of the END [11].

(iii) HA_CS and HSD_CS. The total number of people (N) affected by the harmful effects of level of noise due to road noise exceeding the proposed limits for *L_den_* and *L_night_* and calculated using the dose–response curve estimated in the case-study area.

To allow a correct comparison to analyze the performance of the HSIP 3D tool output, the spatial data of the evaluation points were normalized. The normalization was conducted using the percentage of the indicators per evaluation point regarding the total analyzed length of the highway. It was then possible to compare (pairwise analysis) the distribution along the highway of these 6 different results defined. To compare whether the six community noise assessment indicators define the same sections of highway to prioritize, the following process has been carried out. First, the output of the HSIP 3D-tool was classified by quartiles to later transform the scalar data into binary using the simple rule: if the data evaluation point belongs to Q1 (high priority), it becomes 1; otherwise it becomes zero.

Because the current Ecuadorian legislation does not apply *L_den_* and *L_night_*, the possibility of generating direct transformations between the noise indicators *(L_den_, L_night_, L_dia,_* and *L_noche_)* were explored without the need to generate new noise maps and new analyses. National noise indicators *L_dia_* and *L_noche_* (which are denoted in Spanish so as not to create misunderstandings) consider two reference time intervals. After a revision in 2015, these periods are *L_dia_* (from 07:00 to 21:00 h) and *L_noche_* (from 21:00 to 07:00 h).

#### Statistical Tests for Comparison of HSIP 3D-Tool Output with the Different Noise Indicators

Dynamic time warping (DTW), Spearman‘s correlations coefficient, Kendall’s Concordance, Friedman’s test, Wilcoxon signed-rank test, and clustering analysis were used for the analysis and to look for new insight of the outcome data extracted from the application of the HSIP 3D-tool. SPSS, Matlab, and R were used for the data analysis. These tests will be briefly explained as Supplementary Material File S4.

## 3. Results

The results of the analysis of the responses, the calculation of the dose–response curves, and their comparison are addressed in this section. Later, the hot-spots defined by the six community noise assessment indicators are compared.

### 3.1. Results from the Subjective Data Collected during the Survey Campaign

This subsection includes a brief statistical analysis of the responses given to the questionnaire, the calculation of the dose–response curves for the study area, and the comparison of the dose–response curves of our study with the ones of the WHO.

#### 3.1.1. Descriptive Statistical Analysis of Socio-Acoustic Information

A total of 244 valid questionnaires, filled by people aged between 18 and 80 years old (51.23% men, 48.7% women), were considered in the data analysis. In 69.26% of households, more than three people lived together. Of those interviewed, 96.72% had a direct view of the highway from their living room and 52.87% from their bedroom.

All the interviewees were exposed to *L_den_* higher than the threshold that the WHO considers a health risk (*L_den_* ≥ 53 dB, *L_night_* ≥ 45 dB). The highest percentage of people were exposed to *L_den_* = 74–75 dB range (27.35%). For *L_night_* however, the highest percentage of people were exposed to the 62–65 dB range (21.22% exposed to 62–63 dB and 28.57% exposed to 64–65 dB). More information about the noise levels that participants were exposed to can be found in the Supplementary Materials File S5. If we compare the main questions of the study, we find that the percentage of HA_CS is slightly higher than the percentage HSD_CS and similar in range to other studies [39]. Remarkably, more than 85.25% of interviewees could hear the traffic with windows open, and of them, 90.86% expressed low, moderate, or high annoyance with the traffic noise. The distribution by floor of the population interviewed shows that 68.45% of them lived above the third floor and only 3.69% on the first floor. Further information about the participants’ responses split by the percentage of noise annoyed and sleep-disturbed people can be found in the Supplementary Material File S6.

The distribution regarding the question of annoyance shows that 13.11% of the participants were not annoyed, 25% slightly annoyed, 35.66% moderately annoyed, and 26.23% highly annoyed. Among the participants, 16.8% were not sleep disturbed, 32.38% were slightly sleep disturbed, 31.15% were moderately sleep disturbed, and 19.67% were highly sleep disturbed. It should be noted that the high percentages of people disturbed by noise are because the interviews were conducted with the tenants of the houses that faced directly onto Mariscal Sucre Avenue.

Next, we analyzed the main results obtained related to the correlations between variables. As expected, annoyance is positively correlated with the values of *L_den_* (r = 0.4) and *L_night_* (r = 0.39), with a “medium” association between variables according to Cohen’s criterion [40]. Age is positively correlated with noise annoyance (r = 0.20), which is associated with the fact that older people tend to be more annoyed by noise than the youngest, but this is not correlated with sleep disturbance. It is also inversely correlated with the temporal comparison during the day (r = −0.14), which means that older people tend to think that noise is higher now than before the pandemic. It is also directly correlated with work interference (r = 0.14). Both noise annoyance and the temporal comparison during the day present a “small” strength of association with age. Sleep disturbances are negatively correlated with closing the window during the night because of the traffic noise. The question about closing the window is a yes–no question, with ratings yes = 1, no = 2. Therefore, a negative correlation involves that people with sleep disturbances tend to have to close the window at night because of the noise. As expected, hearing traffic noise with windows open is positively correlated with closing the window because of noise disturbances (r = 0.60), with a “large” association between variables.

The results of the linear regression models calculated show that changes in the predictors are associated with statistically significant changes in noise annoyance and sleep disturbance for all the variables under study, but for the number of people living in the house, they are associated with direct vision from the living room and the bedroom, the floor in which participants live, whether or not they work from home, and the gender. The age in our study, however, explains changes in the annoyance, but not in the sleep disturbance. More information about the results of the correlations between variables and the linear models calculated is shown in the Supplementary Material File S7.

The calculation of Cronbach’s alpha coefficient showed internal consistency and consequently reliability of the questionnaire designed. For example, the internal consistency for the noise annoyance and sleep disturbance was good (Cronbach’s alpha = 0.84, 95% confidence interval [0.79, 0.87]). Furthermore, Cronbach’s alpha for temporal comparisons before and after the pandemic of the sleep disturbance and noise annoyance was 0.66 (95% confidence interval [0.56, 0.73]), which is considered acceptable for exploratory studies [41]. For questions about hearing the noise from Mariscal Sucre Avenue with windows open and closed and having to close the window during the day, the internal consistency was also acceptable (Cronbach’s alpha = 0.67, 95% confidence interval [0.59, 0.73]). Regarding hearing the noise from Mariscal Sucre Avenue and having to close the window at night, the internal consistency was good (Cronbach’s alpha = 0.78, 95% confidence interval [0.72, 0.82]).

#### 3.1.2. Exposure–Response Relationship for Highly Sleep Disturbed and Highly Noise Annoyed for the Case Study Area

A quadratic regression analysis was performed using a generalized linear model to evaluate the exposure–response relationship of the percentage of people highly sleep disturbed and *L_night_* (Table 1). The *L_night_* noise levels were ordered increasingly together with the number of highly sleep-disturbed people (HSD) affected. For each *L_night_*, the average value of the corresponding HSD, and the previous one (HSD*_Lnight_* _XdB_ + HSD*_Lnight_* _x−1dB_)/2 were calculated to reduce the effects of outliers. With these values, the percentage of highly sleep-disturbed people by each dB was calculated. The equation for the %HSD_CS is defined as follows:

%HSD_CS = A * *L_night_*^2^ + B * *L_night_* + C

The total variance in %HSD_CS explained by the model is 91.97% (R^2^ = 0.9197, adjusted R^2^ = 0.8997, *p*-value = 4.15 × 10^−5^).

The linear model %HSD_CS = A * *L_night_* + B was also calculated. Its coefficients, standard errors, and *p*-values are shown in Table 2. The total variance in %HSD_CS explained by the model is 91.70% (R^2^ = 0.9170, adjusted R^2^ = 0.9078, p-value = 3.66 × 10^−6^).

The curvature of the quadratic model is very little pronounced, showing the percentages of HSD_CS with very few differences from those of the linear model for the different noise levels evaluated. These differences are between +0.66% and −0.70%.

Although the coefficient of determination for the linear model is slightly smaller than the one of the quadratic model, the coefficients and intercept are statistically significant (*p*-value <0.05), and the standard error is smaller (Table 1 and Table 2). Consequently, the linear model was selected.

#### 3.1.3. Exposure–Response Relationship for Noise Annoyance

Following the same procedure, a quadratic regression model was calculated for the percentage of highly annoyed people. The coefficients and their performance are shown in Table 3. The equation for the %HA_CS is defined as follows:

%HA_CS = A * *L_den_*^2^ + B * *L_den_* + C

The total variance in %HA_CS explained by the model is 94.38% (R^2^ = 0.9438, adjusted R^2^ = 0.9298, *p*-value = 9.94 × 10^−6^).

The coefficients, standard errors, and *p*-values of the linear model for the HA are shown in Table 4. The total variance in %HA_CS explained by the model is 92.20% (R^2^ = 0.9220, adjusted R^2^ = 0.9133, *p*-value = 2.766 × 10^−6^).

The quadratic model for the prediction of the %HA_CS has a flatter shape at the beginning of the curve (than the linear model), showing 8.42% of HA_CS exposed to 58 dB in comparison with 4.24% for the linear model. At 70 dB and 76 dB, the values of both curves are very similar (14% and 45% approx.): among these noise levels, the values are lower for the quadratic model, with a maximum difference of 2.87% at 73 dB. Above 76 dB, the growth of the quadratic curve is steeper, leading to 59.59% at 78 dB in comparison to 55.42% for the linear model.

Although the quadratic model better explains the variance than the linear model, the last one was preferred because the coefficient and intercept are statistically significant, and the standard error is smaller (Table 3 and Table 4).

**Figure 2:**
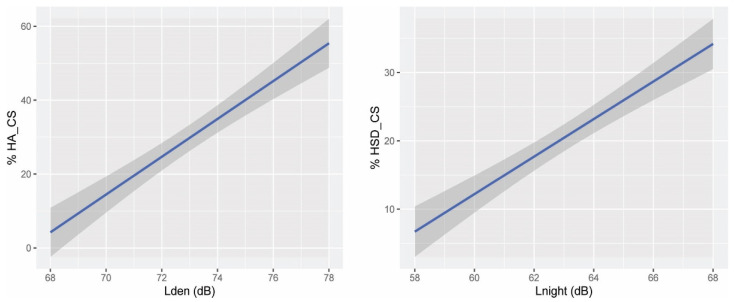
(**Left**): Exposure–response curve for *L_den_*-HA_CS. (**Right**): Exposure–response curve for *L_night_*-HSD_CS.

For both prediction curves %HA_CS and %HSD_CS (Figure 2), only the interval of the curves calculated from real data was represented (68 dB to 78 dB and 58 dB to 68 dB, respectively).

#### 3.1.4. Comparing the Dose–Response Curves for the Percentage of HA_CS and HSD_CS of the Study Area with the Dose–Response Curves of the WHO

**Figure 3:**
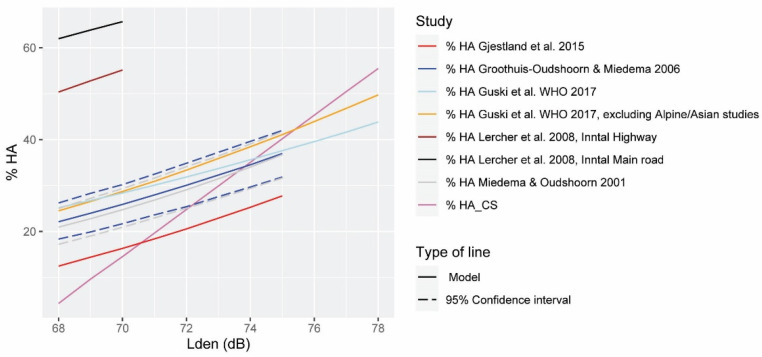
Comparison of the exposure–response curves for the percentage of highly annoyed people according to the full-range studies (light blue), and the full range excluding the Alpine/Asian ones evaluated by Guski et al. [15] (orange), to Gjestland et al. [19] (red), to Groothuis–Oudshoorn and Miedema [43] (blue), Miedema and Oudshoorn [42] (grey), and Lercher et al. [22,44] (brown for highway, and black for main roads), and according to the case study (purple). The prediction models are drawn in solid lines, and the confidence intervals, when available, are in dashed lines.

**Figure 4:**
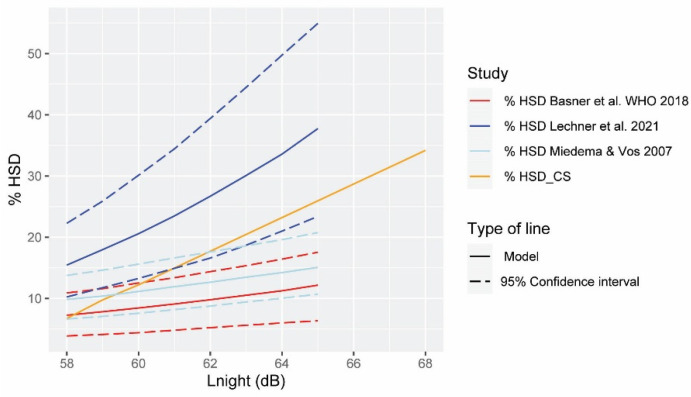
Comparison of the exposure–response curves for the percentage of highly sleep-disturbed people according to Basner et al. [14] (red), Lechner et al. [45] (blue), Miedema and Vos [46] (light blue), and the case study (orange). The prediction models are drawn in solid lines, and the confidence intervals, when available, are in dashed lines.

Hypothesis H1 was formulated to assess whether the dose–response curves for the percentage HA_CS and HSD_CS calculated are within the 95% confidence interval of the dose–response curves of the WHO. For that purpose, Figure 3 and Figure 4 were built for the noise levels range of our study.

Figure 3 shows a comparison of the exposure–response curves for *L_den_*-HA_CS from various research studies, among them, that of Guski et al. conducted for the WHO in 2017 [15]. Guski et al. did not calculate confidence intervals in their study. For this reason, we have compared the results of our study with the ones of Miedema and Oudshoorn of 2001 [42], as their dose–response curve (and its confidence intervals) has been considered as a reference in many WHO reports [3,7], and their curve is very similar to that of Guski et al. Figure 3 shows that the dose–response curves of most of the studies represented end at 75 dB or before (but the dose–response curve of Guski et al.). The curve calculated in our study is within the confidence interval of the Miedema and Oudshoorn only, from 72 dB to 75.5 dB approx. (if we follow the expected growth progression of their dose–response curve). The quadratic model showed similar results, as the curve only fits within the confidence intervals of approx. 73 dB and 75.6 dB. Therefore, hypothesis H1 is not met for the dose–response curve HA calculated for our study area. Further analysis of the comparison of the curves can be found in Section 4: Discussion.

Figure 4 shows the comparison of the *L_night_*-%HSD_CS dose–response curve of our study with different research works, including that of Basner et al. for WHO [14], 2018. The curve of our study is only within the confidence interval of the WHO dose–response curve from approximately 58 dB until 60.2 dB (this is similar to the quadratic model). Therefore, hypothesis H1 is also not fulfilled for the HA dose–response curve calculated for our study area. Further analysis of the comparison of the curves can be found in Section 4: Discussion.

### 3.2. Comparison of the HSIP 3D-Tool Output Data for the Proposed Indicators

This subsection evaluates Hypothesis H2, which was formulated to assess if there are significant differences between the hot-spots calculated using the selected community noise assessment indicator.

**Figure 5:**
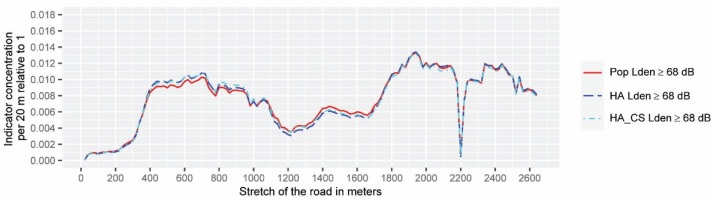
Interclass spatial variation of the community noise assessment indicators referred to the intervention area above *L_den_* ≥ 68 dB. The data are ordered in meters in the ascending direction (from south to north).

**Figure 6:**
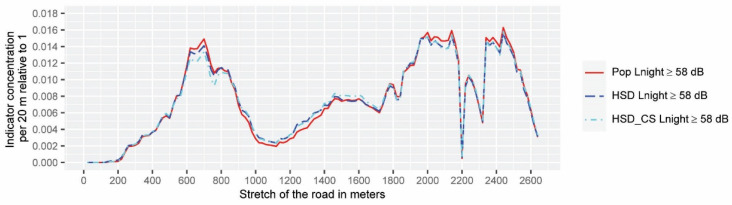
Interclass spatial variation of the community noise assessment indicators referred to the intervention area above *L_night_* ≥ 58 dB. The data are ordered in meters in the ascending direction (from south to north).

The tool provides an output that condenses the information in 1D spatial data that are easy to be analyzed and ready for being graphically represented. The data for statistical tests are those included within the intervention area and will be shown in Figure 5 and Figure 6.

This data corresponds only to a part of the case study area of length 2640 m, sampled by 132 evaluation points (the abscissa represents only the intervention area), and the ordinate axis represents the relative values of each of the indicators expressed in percentages (relative to 1). Statistical tests are applied to this database created from the results of the application of the community noise assessment indicators for different input noise levels (132 × 6 matrix). The analysis is distinguished between inter-class comparisons when comparing different noise assessment indicators based on *L_den_* (the same for *L_nigh_*_t_) using the same noise limit for analysis, and total comparisons, regardless of the selected noise limits and type of indicator.

**Figure 7:**
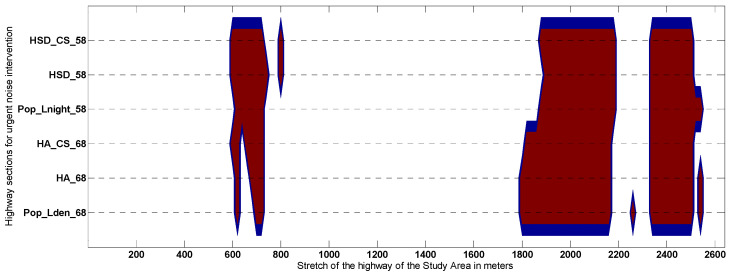
Representing the extension of the highway for short-term intervention using the first quartile. The evaluation points included in the section of the road classified as “urgent” are in red.

Figure 7 represents the data after the transformation, which allows comparing whether the areas of urgent intervention are similar. Remember that at this stage it is intended to make the comparison in the extension of the road classified as “priority intervention area for noise mitigation”, regardless of the levels of the indicators in each of the evaluation points. For this reason, the scalar series are replaced by nominal series according to the scale: 1 (urgent) and 0 (not urgent).

#### Statistical Tests Results

The goal of this section consists of comparing and analyzing the performance of the HSIP 3D-tool output data (six outcomes represented in Figure 5, Figure 6 and Figure 7) using a set of statistical and analytical tests. Strictly speaking, any dwelling subjected to unadvised levels of environmental noise should be considered within the scope of the noise action plan. In this study, noise hot-spots are identified as those evaluation points (within the intervention area) where each community noise assessment indicator has highlighted a greater environmental risk to health. As the test results are quite conclusive, a summary of them is attached, and a more detailed description can be found in the Supplementary Material File S8. To analyze the similarity between the shapes of the data set shown in Figure 5 and Figure 6, two types of tests have been used. Both the DTW analysis and the Spearman correlation analysis point to a very high positive correlation between all indicators (especially inter-class). When testing whether all the community noise assessment indicators rank the evaluation points from highest to lowest importance coincidentally, Kendall’s Coefficient of Concordance is applied, which proves that it tests for a strong agreement in the results. Supporting Kendall’s Coefficient of Concordance results, the Friedman test shows there is not an overall statistically significant difference between the mean ranks of the data shown in Figure 5 and Figure 6. Therefore, all the spatial data output of the HSIP 3D-tool belongs to the same population. Finally, hierarchical cluster analysis for dichotomic data was applied to the dataset shown in Figure 7 to conclude that the extent of priority areas are practically identical when inter-class indicators are analyzed, but when a total comparison is carried out (between the data based on *L_night_* and *L_den_*), differences are found that will be discussed in the following section.

## 4. Discussion

A strategic noise map of a stretch of a major road encircling the city of Quito was developed to serve as a base to carry out this work. The facade noise map of the exposed buildings aids in estimating the population exposed to noise. This information allowed us to plan the survey of the population and, in parallel, to test the one-dimensional spatial data outputs from the HSIP 3D-tool. This area has become an on-site laboratory to test any hypothesis that may arise. To this end, this section will analyze, separately and jointly, both the data obtained from the analysis and statistical processes in GIS, as well as the data from the surveys carried out on the residents of the study area. The research used different kinds of analysis, aiming to determine relation as well as compare results between community response and noise from high traffic highways in a study area of Quito.

### 4.1. Analysis of the Survey Data

The first research question is related to the estimation of the dose–response curves of annoyance and sleep disturbances associated with a major road in Quito and the existence of possible local factors that may affect their shape. In order to discuss the results obtained, the data of the survey and the comparison of the dose–response curves of the case study with the ones of other studies were analyzed.

In behavioral science studies, due to the variability between subjects and the multiple factors that can affect their responses, the correlations between variables are not usually very high [40]. In the study area, the correlation coefficient of the %HA_CS and *L_den_* is 0.4 and between %HSD_CS and *L_night_* is 0.37, which, according to Cohen’s criterion represents a medium association. However, these data are in line with the results of similar studies on noise annoyance and sleep disturbance. For example, Gusky et al. conducted a meta-analysis for the WHO on the basis of the correlation coefficients related to the annoyance caused by different types of environmental noise [15]. For the studies on traffic road noise evaluated in that study, the maximum r correlation between *L_den_* (or *L_dn_*) and %HA was 0.535 [17], and the minimum was 0.004 [17], with a “summary” correlation of 0.325. It is worth highlighting that this correlation and all the others calculated for the present study just show the existence of a linear relationship (positive or negative) between variables, but it is not possible to establish a cause–effect association.

The dose–response curve for the %HA_CS is not within the 95% confidence interval of Miedema and Oudshoorn of 2001 [42]. If we compare the results of the exposure–response curves of our study with the meta-analysis carried out by Guski et al. for the WHO in 2017 [15], we can observe that the curve of our study has a much steeper slope than that of the WHO (Figure 3). On the one hand, it would seem that at a level of up to approximately 73.6 dB, the population in Quito is more tolerant compared to the WHO’s dose–response curve, but much more sensitive as we move away from that value. It is clear that the comparisons cannot be exact, because the WHO dose–response curve encompasses studies of different types of roads and different locations (valleys, highways, main roads, etc.), and the sensitivity to noise varies for each case. Several interesting studies appear in the WHO report for their atypical values of HA compared to the same noise exposure values in other cities. Such is the case in the study conducted in Vietnam [19] (red dose–response curve-Figure 3), with a very small HA for exposures above 65 dB, and a study in Inntal (Austria valley) [15,22,44] with a high HA. In this last study, more participants were more annoyed than those represented in the WHO curve for all the noise levels considered (from 40 dB to 70 dB), both in the curve representing the annoyance caused by the main roads and in the curve that represents the annoyance caused by highways. In the dose–response curves of Inntal, the percentages of very annoyed people are not represented for *L_den_* > 70 dB. However, if we follow the expected progression of the curve towards these values, it seems that the percentage of very annoyed people would be similar to that of our study (Figure 3). It should be noted that in the aforementioned study by Lercher et al. [44], for similar noise levels, the main roads generate a greater degree of annoyance than the highways. In Wipptal, also an alpine valley, the difference between the %HA for main roads and highways can be appreciated, as well as a higher %HA than that of the WHO dose–response curve [15,22,44].

Although Quito is in a valley, and problems similar to those of Inntal or Wipptal can occur, the number of annoyed people experiencing noise levels between 64 and 70 dB is much lower than in these studies (43% less if we compare the Inntal highway and our study at *L_night_* = 70 dB). Furthermore, the fact that there is one section of the curve above and another below the WHO curve may point to factors that are not present in other countries. It should be noted that Quito is a city with few climatic changes throughout the year but with a marked difference in temperature between day and night. During the course of the year, the temperature generally varies from 9 °C at night to 19 °C during the day. It rarely drops below 7 °C or rises above 21 °C. Although the temperature difference between day and night is considerable, the thermal inertia of buildings materials involves the fact that inside the rooms there are no large variations in temperature throughout the day and that good thermal insulation walls are not necessary. This means that the local construction tradition uses enclosure systems with a single layer (solid brick or concrete block with a thickness between 10 and 20 cm), without any type of thermal or acoustic insulation, and windows with a monolithic thin glass (4–8 mm), which is unthinkable in cities with greater temperature changes throughout the year. As there are no regulatory requirements for noise protection inside residential buildings, the acoustic insulation of the enclosures is very low.

Bridging the gap between countries that may exist in acoustic insulation due to the use of different materials and construction systems, in Spain, the Catalog of Constructive Elements [47] establishes a reference value for the masonry of perforated brick (single panel is normally used in Spain for interior separation; 15 cm in total) for the acoustic insulation to traffic noise (RAtr) of approximately 39 dB, compared to the insulation of a traditional double panel of brick masonry (7 cm hollow brick panel + thermal/acoustic insulation + 11.5 cm perforated brick panel, with interior and exterior cladding of the masonry; approximately 25 cm in total) of 45 dB. It is important to highlight that the effect of the windows must be added, which greatly reduces the insulation compared to the insulation of the masonry.

It is worth noting that we reduced the initial number of non-noise-related questions to shorten the length of the questionnaire and achieve greater participation, because some studies suggest that surveys with a large number of questions can result in participants responding with less scrutiny near the end of the questionnaire [48]. However, a large number of questions related to noise may have led to more negative answers regarding noise due to a cumulative effect of the perceived annoyance. Nevertheless, we have not found studies that assess the magnitude of the effect of cumulative noise questions (nor the effect of long questionnaires about annoyance), so this could be a factor to be studied in future research, specifically addressing local differentiating factors.

We think that aspects such as the location (of the study area) in a valley and the poor acoustic isolation of the buildings on the one hand (which might cause a higher %HA), and the possibility that people are more tolerant to noise on the other hand (which might cause a lower %HA), could be the reasons for the steeper slope of the dose–response curve of our study.

The *L_night_*-%HSD_CS dose–response curve has also a steeper slope than the one of the WHO. Above 58.2 dB, the %HSD_CS increases enormously, reaching 28.57% of people highly annoyed for *L_night_* = 64–65 dB, compared to an approximate percentage of 11.20 according to the WHO’s study. However, it is within the confidence intervals of the study by Lechner et al. [45] carried out in Innsbruck, in the Inntal valley, although with approximately 10% fewer people annoyed at 65 dB. In the study by Lechner et al., however, the degree of annoyance is greater than in the curves of the WHO and Miedema and Vos [46] along the entire curve, and therefore, theirs shows a different trend than our study. The reasons could be similar to the ones for the %HA dose–response curve; however, factors that might increase the percentage of HSD (such as location in a valley and poor acoustic isolation) might have a higher weight in the results for the sleep disturbances.

### 4.2. Discussion concerning the Characterization of Noise Hot-Spots on Major Roads

The second research question has to do with the applicability of determining if the local aspects identified have any impact on the definition and quantification of the importance of noise hot-spots on highways with high-density traffic due to their consequences in the design of action plans. This section of the study focuses on two basic aspects.

- Whether there is a deviation between the dose–response indicators proposed by European regulations regarding the local dose–response curves estimated in this research from the study area of Quito;
- To examine the impact that the use of reference time intervals (END vs. National), in combination with the use of dose-response curves (WHO vs. Case Study), may have on global estimates of the number of people exposed to noise in the study area.

The inter-class differences between indicators used to characterize hot-spots are not perceptible, because the evaluation noise limits are very high (68 dB *L_den_* and 58 *L_night_*), and therefore the exposed population distributions have a very small range of noise values. Therefore, it can be affirmed that there are no appreciable differences between the evaluation of HSD and HA proposed by the WHO and those elaborated in this study. There are also no differences between these dose–response indicators when compared directly to the population living above 68 dB *L_den_* and 58 *L_night_*. Additionally, when evaluating the differences between all six community noise assessment indicators, the characterization of the hot-spots is shown to be similar for all of them.

After applying hierarchical cluster analysis for dichotomic data, an appreciably different definition can be stated for the extension of the urgent areas of intervention when comparing indicators based on *L_den_* with those based on *L_night_*. To guarantee the robustness of the conclusions, it was shown that the number of iterations always exceed 25 regardless of the methods for calculating the distances (Euclidean distance, pattern difference, simple matching, and Jaccard) and the selection of the distance measurement method (averaged linkage between groups and within groups and centroids clustering linkage). We suspect that this is due to due to the instability of defining urgency of intervention only using the data associated with the first quartile. In future studies, the definition of these zones should be explored using an algorithm that exploits not only the density of the variable but the contiguity of the candidate evaluation points used to establish the extension and priority of the road sections. This is closer to reality where the action plans are applied to continuous road sections with a minimum extension.

For the second aspect, it would be interesting to create a fast and simple conversion between the *L_den_*–*L_dia_* and *L_night_*–*L_noche_* pairs without the need to generate new noise maps [49]. As most of the existing scientific studies on noise and annoyance have been generated using *L_den_*, conversions between noise indicators can be useful in something as simple as helping environmental authorities to establish noise limits in future legislation.

Very few questions on the test imply the consideration of the reference day period, and then we do not believe it makes sense to convert the dose–response curves created in this study for *L_den_* to *L_dia_*. However, a discussion of this conversion is found in the Supplementary Material File S9. This is not the case for night noise, and the equivalences, in this case, are defined in Table 5.

It can be said that by adding the total traffic during the two hours of the evening period from 21:00 to 23:00 h to the total traffic in the period from 23:00 to 07:00 h the traffic density per hour varies, as does the percentage of heavy vehicles. These statements were analyzed using CADNA in the development of new noise maps in which the population exposed at *L_noche_* was directly calculated, keeping all the parameters of the calculation fixed except for the reference time periods. The distributions of people exposed to night noise are shown in the Supplementary Material File S10. The data for both maps serve for comparison between estimations of the total number N of people at risk of harmful effects due to traffic noise during the nighttime considering different dose–response curves (Table 6).

### 4.3. Limitation of the Study

The validity of the study should be limited to those areas of Quito where residential buildings are highly exposed to noise from major roads (exceeding 68 dB *L_den_* and 58 *L_night_* on the façade of buildings) and with a direct line of sight to the road from bedrooms and living rooms. Also, it will be necessary to understand how objective exposure to traffic noise, subjective annoyance, and the socio-economic dimension of the interviewees are related in Quito, as a prerequisite for the generalization of the findings to other neighborhoods of the city affected by the major road infrastructure.

## 5. Conclusions

Although the survey was carried out in buildings exposed to high noise levels, the results show that the percentages of the highly annoyed people for noise levels ≥ 73.6 dB for *L_den_* and ≥ 58.2 dB for *L_night_* are higher than those of the curves of the WHO. This indicates that sleep disturbances and the resulting consequences for health may be especially important since *L_night_* levels of up to 69 dB are reached in the stretch of the studied highway. This high percentage of annoyed people may be due to construction problems of the buildings caused by an unexpectedly low level of acoustic insulation. A possibly related fact is that the effects of noise levels calculated/measured on the façade in countries with different acoustic insulation of buildings may not be comparable with those of Quito. Although the lower part of the curves for HA and HSD may have been different if the population exposed to lower noise levels had been surveyed, it can be noted that the results obtained for the intermediate noise levels of the present study are significant. Consequently, it is necessary to carry out future studies that relate acoustic insulation with the degree of annoyance and the levels of exposure on the façade in the Ecuadorian population.

The new noise dose–response indicators estimated from the data obtained in the local socio-acoustic survey campaign do not provide significant changes in the identification and ranking of hot-spots after the use of the HSIP 3D-tool compared to the results obtained using WHO dose–response curves for *L_den_* ≥ 68 dB for and for *L_night_* ≥ 58 dB. Regarding the prioritization of areas for urgency of intervention, the interclass analysis confirms relevant differences between the definition of the extension of the urgent areas based on *L_den_* ≥ 68 dB and *L_night_* ≥ 58 dB.

The reference time periods for the environmental noise in Ecuador introduce a penalty of +2.2 dB for *L_noche_* with respect to *L_night_.* This should be taken into account when implementing acoustic quality objectives for a diversity of noise zoning (especially for residential areas) based on night noise maps from road traffic.

## Figures and Tables

**Table 1:** Quadratic regression model for predicting the percentage of highly sleep-disturbed people.

	Coefficient	Std. Error	*t* Value	*p*-Value
Intercept	59.88 (C)	407.80	0.147	0.887
*L_night_*	−4.02 (B)	12.97	−0.31	0.765
*L_night_* ^2^	0.05 (A)	0.10	0.55	0.616

**Table 2:** Linear regression model for predicting the percentage of highly sleep-disturbed people.

	Coefficient	Std. Error	*t* Value	*p*-Value
Intercept	−152.65 (B)	17.38	−8.78	1.04 ×10 ^−5^
*L_night_*	2.75 (A)	0.28	9.97	3.66 × 10^−6^

**Table 3:** Quadratic regression model for predicting the percentage of highly annoyed people.

	Coefficient	Std. Error	*t* Value	*p*-Value
Intercept	1156.80 (C)	851.00	1.36	0.211
*L_den_*	−36.07 (B)	23.35	−1.55	0.161
*L_den_* ^2^	0.28 (A)	0.16	1.77	0.116

**Table 4:** Linear regression model for predicting the percentage of highly annoyed people.

	Coefficient	Std. Error	*t* Value	*p*-Value
Intercept	−343.74 (B)	36.25	−9.48	5.56 × 10^−6^
*L_den_*	5.12 (A)	0.50	10.31	2.77 × 10^−6^

**Table 5:** Equivalence between National and END noise indicators.

Indicator to Estimate	The Output of Noise Mapping	Average Traffic Flow per Hour during the Nighttime as a Percentage of Total Traffic	National and END Noise Indicator Equivalences
*L_noche_*	*L_night_*	21:00–07:00 h, 1.8% v/h (noche) 23:00–07:00 h, 1.1% v/h (night)	*L_noche_* ≈ *L_night_* + 2.2 dB

**Table 6:** HSD (High sleep disturbance) was calculated based on the dose–response curves for the case study and HSD based on WHO.

HSD_CS Strategic Noise Map Results (*L_noche_* ≥ 60 dB)	HSD_CS Strategic Noise Map Results (*L_night_* ≥ 58 dB)	HSD Strategic Noise Map Results (*L_night_* ≥ 58 dB)
Total number N of people at risk of a harmful effect due to traffic noise		
189 people out of a total of 1574	159 people out of a total of 1543	148 people out of a total of 1543

## Data Availability

The data that support the findings of this study are available on request from the corresponding author.

## Supplementary Materials

The following are available online at https://www.mdpi.com/article/10.3390/ijerph19031115/s1, File S1. Façade noise maps and estimation of the number of people exposed to noise; File S2. Main questions of the survey; File S3. The tool for detection and hierarchization of hot-spots (HSIP 3D-tool); File S4. Statistical tests used for the data analysis; File S5. Percentages of interviewed people exposed to the different noise levels; File S6. Table of characteristics of the interviewees and their home environment; File S7. Table of results of the linear models for annoyance and sleep disturbance; File S8. Statistical test results of the different dose–response curves; File S9. Conversion between *L_den_* and *L_dia_*; File S10. Results of the maps’ exposition for *L_night_* and *L_noche_*.
