# Supplementary material for: Community Response to Noise from Hot-Spots at a Major Road in Quito (Ecuador) and Its Application for Identification and Ranking These Areas
Source: Int J Environ Res Public Health. 2022 Jan 20;19(3):1115. doi: 10.3390/ijerph19031115 (PMC8834050; doi:10.3390/ijerph19031115)
Supplement: Supplementary file 1 [file ijerph-19-01115-s001.zip › ijerph-1484788-supplementary.pdf]

International Journal of
Environmental Research and Public Health

MDPI

Supplementary Materials: Community Response to Noise from Hot-Spots at a Major Road in Quito (Ecuador) and Its Application for Identification and Ranking These Areas

Virginia Puyana-Romero [1,*], Jose Luis Cueto [2], Giuseppe Ciaburro [3], Luis Bravo-Moncayo [1] and Ricardo Hernandez-Molina [2]

1 Grupo de Investigación Entornos Acústicos, Departamento de Ingeniería en Sonido y Acústica, Campus Granados, Universidad de Las Américas, Quito 170125, Ecuador; luis.bravo@udla.edu.ec
2 Laboratorio de Ingeniería Acústica, Campus de Puerto Real, Universidad de Cádiz, 11510 Puerto Real, Spain; joseluis.cueto@uca.es (J.L.C.); ricardo.hernandez@uca.es (R.H.-M.)
3 Dipartimento di Architettura e Disegno Industriale, Università degli Studi della Campania "Luigi Vanvitelli", Borgo San Lorenzo, 81031 Aversa, Italy; giuseppe.ciaburro@unicampania.it
* Correspondence: virginia.puyana@udla.edu.ec

Citation: Puyana-Romero, V.; Cueto, J.L.; Ciaburro, G.; Bravo-Moncayo, L; Hernandez-Molina, R. Community Response to Noise from Hot-Spots at a Major Road in Quito (Ecuador) and Its Application for Identification and Ranking These Areas. *Int. J. Environ. Res. Public Health* **2022**, *19*, 1115. https://doi.org/10.3390/ijerph19031115

Academic Editors: Antonio J. Torija Martinez and Paul B. Tchounwou

Received: 13 November 2021
Accepted: 17 January 2022
Published: 20 January 2022

Publisher's Note: MDPI stays neutral with regard to jurisdictional claims in published maps and institutional affiliations.

Copyright: © 2022 by the authors. Submitted for possible open access publication under the terms and conditions of the Creative Commons Attribution (CC BY) license (https://creativecommons.org/licenses/by/4.0/).

File S1. Façade noise maps and estimation of the number of people exposed to noise

In order to obtain consistent data on the most exposed population in which to conduct the survey, a façade noise map of the study area was calculated. This map allowed us to understand the spatial distribution of the traffic noise levels to which the population is exposed. To use the façade noise map for estimation, the road model NMPB-08, implemented in CADNA 2020 was used. This software was used to calculate the A-weighted sound pressure level (L_{Aeq}) in every receiver at the façade of residential buildings. The distribution of these receivers on all the floors of buildings was made according to the German Calculation Method for the Determination of the Number of Persons Exposed to Environmental Noise (VBEB) [1]. The temporal reference periods for the estimation of L_{den} and L_{night} are the usual day from 07 to 19h, the evening from 19 to 23h, and the nighttime from 23 to 07h.

Estimations about traffic flow and composition of the fleet were made using on-site traffic counts during one week, distinguishing between day, evening, and night. In this manner, data were recorded on the AADT (Annual Average Daily Traffic), taking into account two categories: heavy-duty vehicles (HDV) and light vehicles (LV). Figure S1 shows the percentage of the total traffic density per hour. The AADT in the stretch of the avenue under study during the noise and traffic-counting campaigns was estimated in 105,000 vehicles. Sixty-nine percent of the total traffic (approximately 6.000 v/h with 7% of Heavy Traffic) was measured during the daytime hours (06h to 18h), twenty-two percent (approximately 5.800 v/h with 6% of Heavy Traffic) during the evening (18h to 22h) and nine percent (approximately 1.200 v/h with 2% of Heavy Traffic) during the nighttime (22h to 06h).

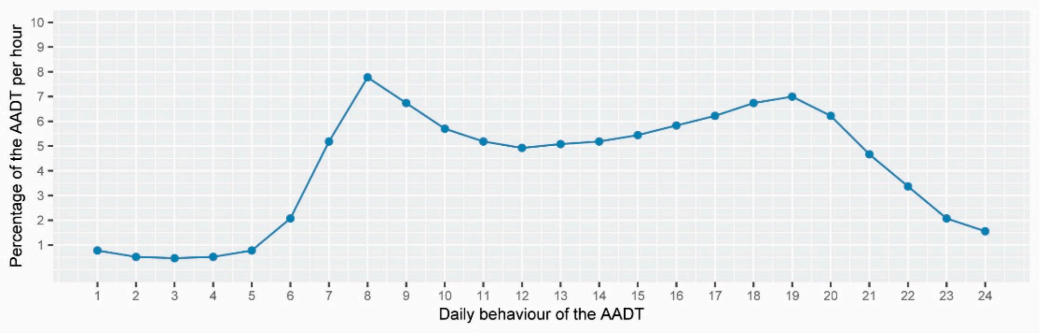

Figure S1. The traffic behavior during 24 h in the studied highway. The data encoded in hour 12 are the value of the traffic in the period of 11-12 h.

Traffic in the study area is normally fluid, and the speed of vehicles (heavy and light) is around 80 km/h (speed limit of 90 km/h for light vehicles and 70 km/h for heavy vehicles). The occurrence of favorable sound propagation conditions is higher than expected due to the temperature inversions during the whole day, and this is reflected in the model parameters.

With the data of people living in the residential buildings and the results of the facade noise maps, CADNA provides the number of people exposed to the noise levels L_{night} and L_{den}. The percentages of people exposed to each noise level with respect to those exposed to L_{night}>53 dB or L_{day} >45 dB are shown in Figures S3 and S4, respectively.

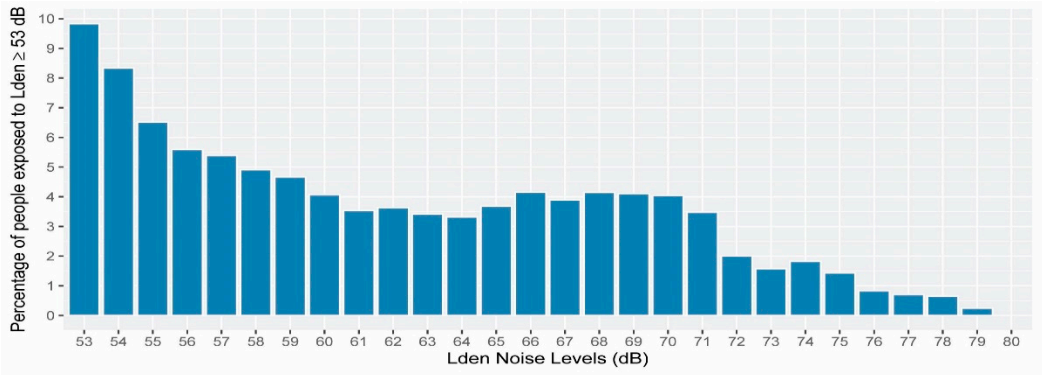

Figure S2. The total number of people living in the mapped area who are exposed in their homes to noise at night that exceeds 45 dB is 5273 persons. The percentage of these people is distributed in the figure in 1 dB classes.

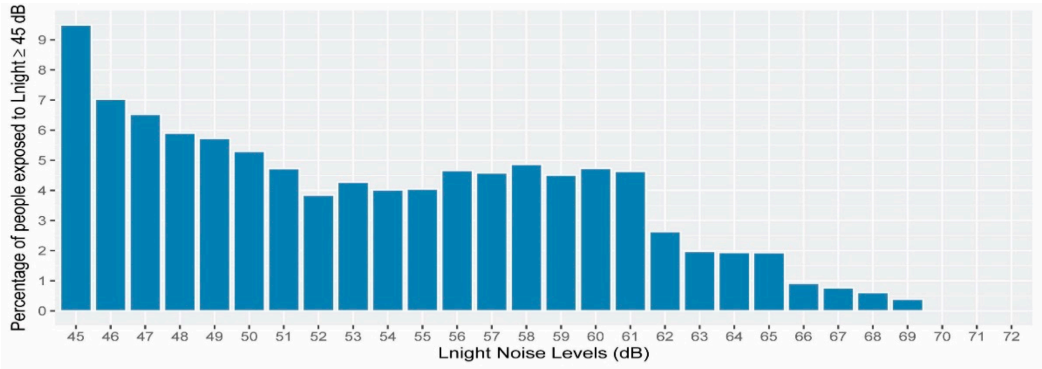

Figure S3. The total number of people living in the mapped area who are exposed in their homes to L_{den} that exceeds 53 dB is 5984 persons. The percentage of these people is distributed in the figure in 1 dB classes.

Finally, with the distribution of the population exposed to different noise levels (Figures S3 and S4), the percentages of annoyance and sleep disturbances were calculated and are reported in Table S1.

Table S1. Summary of the global results from the noise mapping process [2], referring to the total number and percentage of people affected by harmful effects (HA or HSD) of traffic noise within the case study area. The percentage of people at risk of HA or HSD was calculated with respect to the total number of people in the study area exposed to L_{den} > 53 dB or L_{night} > 45 dB, respectively.

**High annoyance (HA)**		**High sleep disturbance (HSD)**	
Strategic noise map indicator (L_{den})		Strategic noise map Indicator (L_{night})	
Total number N of people at risk of	Percentage of people at risk of harmful effects of traffic	Total number N of people at risk of	Percentage of people at risk of harmful effects of traffic

harmful effects of traffic noise	noise for L_{den} greater than 53 dB	harmful effects of traffic noise	noise for L_{night} greater than 45 dB
1097 of a total of 5273 persons	18%	315 of a total of 5984 persons	6%

File S2. Main questions of the survey

Table S2. Main questions of the first, second, and third parts of the questionnaire.

Question (referring to one-year period)	Measurement scale
1. Can you see Mariscal Sucre Avenue when you look out of the living room window of your home?	Yes/no question
2. Can you see Mariscal Sucre Avenue when you look out of the bedroom window of your home?	Yes/no question
3. How much has traffic noise from Mariscal Sucre Avenue annoyed or disturbed you at home?	5 points Likert scale
4. Did you have to work or telework at home due to the pandemic?	Yes/no question
5. If you have answered affirmatively to the previous question, how much has the noise from Mariscal Sucre Avenue interfered with your work?	5 points Likert scale
6. How do you think the traffic noise from Mariscal Sucre Avenue in your home is like today compared to before the pandemic?	5 points Likert scale
7. Is there any type of noise that bothers you more frequently or more intensely than the noise from Mariscal Sucre Avenue in your home?	"No" answer, and 5 options: noise from the neighborhood, animals, overflying planes, noise from other streets, and other noises
8. Has any of the people who live with you complained about the noise produced by Mariscal Sucre Avenue in their home?	Yes/no question
9. How much did traffic noise from Mariscal Sucre Avenue alter your sleep at night?	5 points Likert scale
10. How do you think the traffic noise coming from Mariscal Sucre Avenue at night is currently if you compare it with before the pandemic?	5 points Likert scale
11. Have you heard traffic noise from Mariscal Sucre Avenue in your home during ...	
1. the day, with windows open?	Yes/no question
2. the day, with windows, closed?	Yes/no question
3. the night, with the windows open?	Yes/no question
4. the night, with the windows, closed?	Yes/no question
12. Have you ever had to close the window because the noise from Mariscal Sucre Avenue bothered you ...	

5.	during the day?	Yes/no question
6.	overnight?	Yes/no question

File S3. The tool for detection and hierarchization of hot-spots (HSIP 3D-tool)

The present research uses a 3D GIS-based tool for the identification and prioritization of hot-spots on major roads. An extensive description of the tool applied is discussed in the research article [3]. Before proceeding further, it is worth emphasizing several aspects of the operation of the tool. First of all, this GIS tool is not a noise prediction algorithm, although all the operations it performs are based on noise propagation. On the contrary, the noise data input used by the tool are provided by the strategic noise map, in particular, the data from façade noise maps. In this context, this GIS tool connects noise receivers on the facade of residential buildings (in a 3D scenario) only with the section or sections of the road that directly influence(s) the noise problem detected in each noise receiver. In other words, each noise receiver is linked to a specific section of the road that is the cause of its excess noise (S-R noise Source-Receiver pair). The basic data to handle are the distribution of the number of people exposed to noise levels on all the receivers that exceed the noise limit established by law or any recommendation. From these raw data, it is possible to estimate any previously programmed noise indicator. The noise receivers are the points associated with all the floors of the façades of residential buildings in which the number of people exposed to noise is estimated from the strategic noise maps. Then, the information available in the receivers (R) is transferred, processed, and stored in the partner sections of the road (S) and vice versa. Repeating these tasks iteratively (establishing all S–R pairs, sharing the data between them, processing the data to create new information, etc.) is a very complex process (and time burdensome computationally speaking), but in return it provides a very easy output for decision-makers to handle and interpret. For simplicity, from here on, we will refer to this tool as the HSIP 3D-tool (Hot-Spot Identification and Prioritization).

A peculiarity of the HSIP 3D-tool is that when evaluating a receiver with an L_{night} of (e.g.) 60 dB, it does not define the same road section length when L_{night}=45 dB as when the L_{night}=55 dB limit is introduced. Unsurprisingly, the section of the road that causes the noise on that particular receiver to exceed 45 dB is much longer than the section that causes it to exceed 55 dB. Continuing with the example and as explained above, the data stored at this receiver (number of persons exposed to L_{night}≥60 dB) are transferred only to the section or sections of the road responsible for the noise excess [1].

File S4. Statistical tests used for the data analysis

Various tests were used for the analysis and compression of the outcome data extracted from both the survey and the application of the HSIP 3D-tool. What tests they are and their use will be briefly explained below.

a. For the survey:

(i) Analyzing the internal consistency of the data

The internal consistency of the data for the scales used was evaluated with Cronbach’s alpha using R-package “psych” [8].

(ii) Analyzing the correlation using Spearman

The Spearman correlation coefficient [9],[10] is a nonparametric measure of the strength and direction of association that exists between two variables. The test is used for data that have failed the assumptions of normality necessary for conducting Pearson's correlation. For the survey data, Spearman correlations were calculated to evaluate linear relationships between ordinal and dichotomous variables using the R-package “corrplot” [11]. The arrangement of the variables in the graph used for showing the correlations pertains to the execution of the “hclust” method of grouping variables in the R-package “corrplot”. Cohen's criterion was applied to evaluate the strength of the association between

two variables. According to the aforementioned criterion, correlation coefficients between 0.1 and 0.3 show a "small" association, between 0.3 and 0.5 "moderate", and higher than 0.5 "large".

Linear regression models were also calculated for annoyance and high sleep disturbance as dependent variables and the most remarkable perceptual factors (obtained from the survey) as independent variables.

(iii) Calculating of the dose-response curves: quadratic and linear regression models

Two quadratic and two linear regression models were calculated for the prediction of the percentage of highly annoyed (HA) and highly sleep-disturbed (HSD) people using the R-package "stats" [12]. The function geom_smooth was used to draw the 95% confidence level interval for the predictions of the quadratic models. The performance of both types of models was assessed to select the dose–response curve that better fit the available data. For that purpose, the proportion of the variance for the %HA (dependent variable) that is explained by L_{night} (independent variable) was evaluated using the Coefficient of Determination (R^2). A similar method was used for HSD and L_{den}.

b. For the comparison of the hot-spots calculated with different indicators.

(iv) Analyzing the correlation using Spearman

For HSIP 3D tool output data, SPSS and R were used. Spearman correlation is employed to measure the association between two ranked variables, and it is applied was our study to understand whether the data are sorted in a similar (descending) order. In other words, the test could be used to compare the consistency of the spatial distribution of the noise critical evaluation points between indicators.

(v) Analyzing the similarity and distances between spatial vector shapes using DTW

The first option for a similarity measure would be to rely on the calculation of Euclidean distance using the paired evaluation points of the two series to compare. However, it is sometimes necessary to use other distance measure techniques that are more robust and less sensitive to space shifting. For this purpose, is interesting to explore warping techniques. Dynamic time warping or DTW [13],[14] (it is preferred not to rename the technique as DSW dynamic spatial warping so as not to create confusion) is a classical option for computing the shaped-based similarity between two data series. DTW uses an "elastic" procedure that maps all the warping paths between the points of the pair of series and selects the one that yields a minimum distance between the two series to compare. DTW is often restricted to mapping evaluation points within a sliding window. In the case of this paper, a restricted warping path was used, using a threshold of 60 m forward and 60 m backward. The DTW algorithm used in this study was implemented using MATLAB.

(vi) Paired comparison of the indicators after ranking by Kendall's Concordance

The statistical method, Kendall's Concordance Coefficient W [15], was executed in SPSS to determine whether, after ordering the set of output data (HSIP 3D tool applied with the specification of Table S3) from the highest to the lowest values (descending order), there are significant statistical differences between them. This test assesses the consistency for the combination of noise indicators and noise limits, identifying the same importance (score) of noise problems in every spatial unit. If the indicator output does not agree at all, then it is necessary to analyze their conclusions and consider which indicator is the best for the purpose. Kendall's Coefficient of Concordance expresses the agreement or disagreement in a single number. Kendall's W ranges from 0 to 1, where W= 1 indicates that all judges have ranked the hot spot in the same order.

(vii) Friedman test

In addition, a Friedman test [16] was used to test the differences between groups of indicators as the dependent variable being measured is ordinal. If necessary, post hoc analysis with Wilcoxon signed-rank tests was conducted with a Bonferroni correction.

(viii) Clustering analysis

Clustering analysis was applied to highlight if the six noise-evaluation indicators identified the same areas of urgent intervention. Clustering is a data-processing technique designed to reveal similar characteristics between data by grouping them together [14],[17],[18]. In this case, an agglomerative hierarchical clustering tool was implemented in SPSS to detect those groups of indicators that cluster together. It generates a series of models with a wide range of cluster solutions ranging from 1 (all cases in one cluster) to S (each case is an individual cluster). This means that the procedure is iterative, starting the process by treating each indicator data set as an independent group until the process reaches the situation in which all of them cluster together. The variable used in the analysis is binary.

File S5. Percentages of interviewed people exposed to the different noise levels

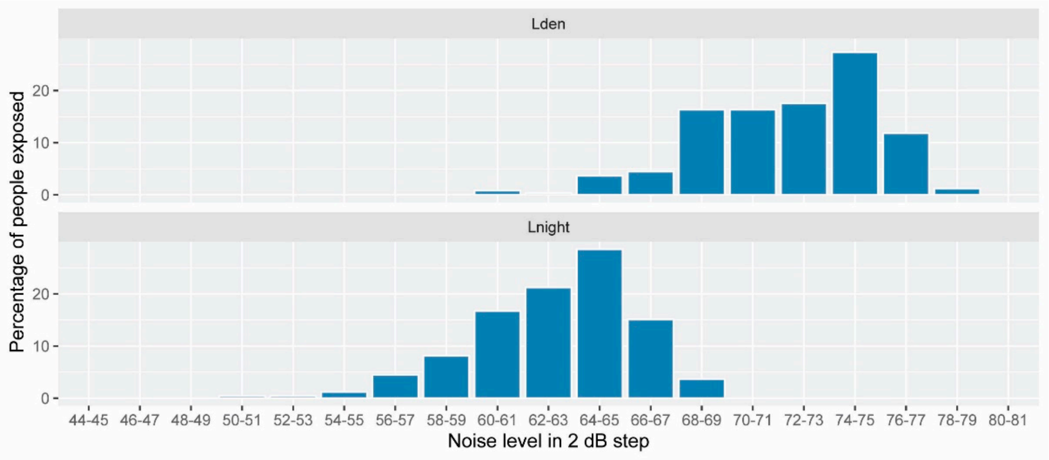

Figure S4. Percentage of interviewed people exposed to L_{night} and L_{den} over 44 dB in 2 dB steps.

File S6. Table of characteristics of the interviewees and their home environment

Table S3 shows a summary of the participants' responses split by the people with noise annoyance and people with sleep disturbance. Between parentheses is the item number of parts two and three of the questionnaire.

Table S3. Characteristics of the interviewees and their home environment, split by the number of people with noise annoyance and sleep disturbance. NNA=not annoyed, L-MA=low or moderately annoyed, HA=highly annoyed, NSD=not sleep disturbed, L-MSD=low or moderately sleep disturbed, and HSD=highly sleep disturbed.

			**People noise annoyed (total and %)**			**People sleep disturbed (total and %)**		
		Total number	NNA	L-MA	HA	NSD	L-MSD	HSD
Gender	Male	125 (51.23)	20 (8.2)	74 (30.33)	31 (12.7)	22 (9.02)	86 (35.25)	17 (6.97)
	Female	119 (48.36)	12 (4.92)	74 (30.33)	33 (13.52)	19 (7.79)	69 (28.28)	31 (12.7)
Age	18-29	88 (36.07)	14 (5.74)	59 (24.18)	15 (6.15)	17 (6.97)	59 (24.18)	12 (4.92)
	30-39	39 (15.98)	3 (1.23)	22 (9.02)	14 (5.74)	3 (1.23)	23 (9.43)	13 (5.33)
	40-49	43 (17.62)	6 (2.46)	27 (11.07)	10 (4.1)	8 (3.28)	26 (10.66)	9 (3.69)
	50-59	56 (22.95)	9 (3.69)	28 (11.48)	19 (7.79)	13 (5.33)	34 (13.93)	9 (3.69)
	>=60	18 (7.38)	0 (0)	12 (4.92)	6 (2.46)	0 (0)	13 (5.33)	5 (2.05)
People living in the house	1	2 (0.82)	0 (0)	1 (0.41)	1 (0.41)	0 (0)	1 (0.41)	1 (0.41)
	2	25 (10.25)	4 (1.64)	13 (5.33)	8 (3.28)	8 (3.28)	11 (4.51)	6 (2.46)
	3	48 (19.67)	11 (4.51)	21 (8.61)	16 (6.56)	10 (4.1)	27 (11.07)	11 (4.51)
	4	92 (37.7)	11 (4.51)	63 (25.82)	18 (7.38)	17 (6.97)	61 (25)	14 (5.74)
	5	69 (28.28)	6 (2.46)	43 (17.62)	20 (8.2)	6 (2.46)	48 (19.67)	15 (6.15)
	More	8 (3.28)	0 (0)	7 (2.87)	1 (0.41)	0 (0)	7 (2.87)	1 (0.41)

Direct vision from living	Yes	236 (96.72)	32 (13.11)	143 (58.61)	61 (25)	41 (16.8)	147 (60.25)	48 (19.67)
	No	8 (3.28)	0 (0)	5 (2.05)	3 (1.23)	0 (0)	8 (3.28)	0 (0)
Direct vision from bedroom	Yes	129 (52.87)	13 (5.33)	80 (32.79)	36 (14.75)	18 (7.38)	85 (34.84)	26 (10.66)
	No	115 (47.13)	19 (7.79)	68 (27.87)	28 (11.48)	23 (9.43)	70 (28.69)	22 (9.02)
Temporal comparison-Day (I6)	Much higher now	5 (2.05)	1 (0.41)	1 (0.41)	3 (1.23)	1 (0.41)	2 (0.82)	2 (0.82)
	A little bit higher	68 (27.87)	3 (1.23)	33 (13.52)	32 (13.11)	3 (1.23)	36 (14.75)	29 (11.89)
	Equal	115 (47.13)	19 (7.79)	78 (31.97)	18 (7.38)	26 (10.66)	77 (31.56)	12 (4.92)
	A little less now	48 (19.67)	4 (1.64)	34 (13.93)	10 (4.1)	8 (3.28)	35 (14.34)	5 (2.05)
	A lot less now	8 (3.28)	5 (2.05)	2 (0.82)	1 (0.41)	3 (1.23)	5 (2.05)	0 (0)
Other sources (I7)	No	40 (16.39)	18 (7.38)	21 (8.61)	1 (0.41)	19 (7.79)	19 (7.79)	2 (.0.82)
	Neighborhood	112 (45.9)	9 (3.69)	66 (27.05)	37 (15.16)	16 (6.56)	66 (27.05)	30 (12.3)
	Animals	112 (45.9)	8 (3.28)	68 (27.87)	36 (14.75)	13 (5.33)	67 (27.46)	32 (13.11)
	Aircrafts	27 (11.07)	3 (1.23)	12 (4.92)	12 (4.92)	3 (1.23)	15 (6.15)	9 (3.69)
	Traffic other	99 (40.57)	5 (2.05)	53 (21.72)	41 (16.8)	6 (2.46)	60 (24.59)	33 (13.52)
	Others	22 (9.02)	8 (3.28)	9 (3.69)	5 (2.05)	5 (2.05)	16 (6.56)	1 (0.42)
Family complaints (I8)	Yes	186 (76.23)	15 (6.15)	118 (48.36)	53 (21.72)	18 (7.38)	130 (53.28)	38 (15.57)
	No	58 (23.77)	17 (6.97)	30 (12.3)	11 (4.51)	23 (9.43)	25 (10.25)	10 (4.1)
Temporal comparison-Night (I10)	Much higher now	3 (1.23)	1 (0.41)	0 (0)	2 (0.82)	1 (0.41)	0 (0)	2 (0.82)
	A little bit higher	51 (20.9)	0 (0)	22 (9.02)	29 (11.89)	1 (0.41)	19 (7.79)	31 (12.7)
	Equal	126 (51.64)	25 (10.25)	77 (31.56)	24 (9.84)	33 (13.52)	82 (33.61)	11 (4.51)
	A little less now	51 (20.9)	5 (2.05)	39 (15.98)	7 (2.87)	5 (2.05)	42 (17.21)	4 (1.64)
	A lot less now	13 (5.33)	1 (0.41)	10 (4.1)	2 (0.82)	1 (0.41)	12 (4.92)	0 (0)
Hear-Day-W. Open (I11-1)	Yes	208 (85.25)	19 (7.79)	126 (51.64)	63 (25.82)	28 (11.48)	135 (55.33)	45 (18.44)
	No	36 (14.75)	13 (5.33)	22 (9.02)	1 (0.41)	13 (5.33)	20 (8.2)	3 (1.23)
Hear-Day-W. Closed (I11-2)	Yes	103 (42.21)	6 (2.46)	40 (16.39)	57 (23.36)	10 (4.1)	51 (20.9)	42 (17.21)
	No	141 (57.79)	26 (10.66)	108 (44.26)	7 (2.87)	31 (12.7)	104 (42.62)	6 (2.46)
Hear-Night-W. Open (I11-3)	Yes	152 (62.3)	12 (4.92)	83 (34.02)	57 (23.36)	12 (4.92)	96 (39.34)	44 (18.03)
	No	92 (37.7)	20 (8.2)	65 (26.64)	7 (2.87)	29 (11.89)	59 (24.18)	4 (1.64)
Hear-NIght-W. Closed (I11-4)	Yes	72 (29.51)	6 (2.46)	26 (10.66)	40 (16.39)	4 (1.64)	35 (14.34)	33 (13.52)
	No	172 (70.49)	26 (10.66)	122 (50)	24 (9.84)	37 (15.16)	120 (49.18)	15 (6.15)
Close w.-Day (I12-1)	Yes	188 (77.05)	10 (4.1)	115 (47.13)	63 (25.82)	21 (8.61)	121 (49.59)	46 (18.85)
	No	56 (22.95)	22 (9.02)	33 (13.52)	1 (0.41)	20 (8.2)	34 (13.93)	2 (0.82)
Close w.-Night (I11-2)	Yes	133 (54.51)	9 (3.69)	71 (29.1)	53 (21.72)	11 (4.51)	78 (31.97)	44 (18.03)
	No	111 (45.49)	23 (9.43)	77 (31.56)	11 (4.51)	30 (12.3)	77 (31.56)	4 (1.64)
Floor	1	9 (3.69)	1 (0.41)	6 (2.46)	2 (0.82)	0 (0)	8 (3.28)	1 (0.41)
	2	36 (14.75)	10 (4.1)	22 (9.02)	4 (1.64)	13 (5.33)	22 (9.02)	1 (0.41)
	3	24 (9.84)	4 (1.64)	13 (5.33)	7 (2.87)	6 (2.46)	14 (5.74)	4 (1.64)
	4 a 8	147 (60.25)	15 (6.15)	89 (36.48)	43 (17.62)	19 (7.79)	93 (38.11)	35 (14.34)
	9 a 13	20 (8.2)	2 (0.82)	12 (4.92)	6 (2.46)	2 (0.82)	13 (5.33)	5 (2.05)

File S7. Table of results of the linear models for annoyance and sleep disturbance

Table S4 shows a summary of the participants' responses split by the people with noise annoyance and people with sleep disturbance.

Table S4. P-value of the linear regression models for annoyance and sleep disturbance.

**Variable**	**Models for Annoyance**	**Models for Sleep disturbance**
	P-value	P-value

Gender	0.451 (f=0.57)	0.253 (f=1.31)
Age	0.001 (f=10.36)	0.156 (f=2.03)
People living in the house	0.526 (f=0.40)	0.349 (f=0.88)
Direct vision from living room	0.294 (f=1.11)	0.810 (f=0.06)
Direct vision from bedroom	0.241 (f=1.38)	0.191 (f=1.72)
Noise annoyance	-	0.000 (f=257.3)
Sleep disturbance	0.000 (f=257.30)	-
Work from home	0.193 (f=1.71)	0.230 (f=1.45)
Noise interference on work	0.000 (f=39.87)	0.000 (f=23.4)
Temporal comparison-Day	0.000 (f=22.82)	0.000 (f=35.95)
Other sources	0.000 (f=20.40)	0.000 (f=19)
Family complaints	0.000 (f=20.36)	0.000 (f=18.18)
Temporal comparison-Night	0.000 (f=19.04)	0.000 (f=26.58)
Hear-Day-W. Open	0.000 (f=39.03)	0.000 (f=19.35)
Hear-Day-W. Closed	0.000 (f=82.68)	0.000 (f=44.33)
Hear-Night-W. Open	0.000 (f=36.26)	0.000 (f=52.46)
Hear-NIght-W. Closed	0.000 (f=27.17)	0.000 (f=36.25)
Close w.-Day	0.000 (f=69.21)	0.000 (f=34.84)
Close w.-Night	0.000 (f=36.7)	0.000 (f=45.17)
Floor	0.816 (f=0.054)	0.500 (f=0.46)

File S8. Statistical tests results of the different dose-response curves

The goal of this section consists in comparing and analyzing the performance of the HSIP 3D-tool output data (six outcomes represented in Figures S8–S10) using a set of statistical and analytical tests.

(i) Analyzing the similarity between spatial vector shapes of the dataset

This test was used to measure the distance between the series included in the dataset in order to estimate the degree of similarity between variables. The DTW algorithm was implemented, which estimated the distance between paired samples of the six noise indicators with a search depth p = ±60 meters with the following results.

Table S5. DTW distance results comparing all the possible combinations of indicators and noise levels. The darker the color, the lower the similarity.

	Pop_Lden_68	HA_68	HA_CS_68	Pop_Lnght_58	HSD_58	HSD_CS_58
Pop_Lden_68	0					
HA_68	0.026	0				
HA_CS_68	0.027	0.022	0			
Pop_Lnight_58	0.182	0.181	0.187	0		
HSD_58	0.207	0.204	0.212	0.0425	0	
HSD_CS_58	0.185	0.181	0.189	0.0256	0.034	0

The greatest dissimilarities (highlighted in dark in Table S5) are found when comparing the noise evaluation indicators based on L_{den} with those based on L_{night}.

(ii) Correlation analysis between the spatial vectors of the dataset

H_o = There is no relationship of the HSIP 3D-tool output data after testing all the combinations of noise evaluation indicators.

A Spearman's rank-order correlation was run to determine the relationship (two by two) between the results of the six different indicators with one each other. Correlations are statistically significant at p-values << 0.01 (two-tailed).

Table S6. Spearman correlation results that compare all the possible combinations of indicators and noise levels. The darker the color, the lower the correlation.

	Pop_Lden_68	HA_68	HA_CS_68	Pop_Lnght_58	HSD_58	HSD_CS_58
Pop_Lden_68	1.000					
HA_68	0.997	1.000				
HA_CS_68	0.995	0.998	1.000			
Pop_Lnight_58	0.856	0.851	0.858	1.000		
HSD_58	0.864	0.861	0.868	0.996	1.000	
HSD_CS_58	0.859	0.856	0.865	0.997	0.998	1.000

There was a strong, positive correlation between results of different indicators per evaluation point, indicating that the null hypothesis—which implies the lack of relationship between all the indicators—should be discarded with more than 99% confidence. In addition, results show a high association (just the vectors that have the same spatial shape) between indicators. The lower levels of correlation were detected when comparing the noise evaluation indicators based on L_{den} with those based on L_{night} (highlighted in dark in Table S6).

(iii) Comparing the coincidence when ordering (rank) from highest to lowest the values of the spatial vectors of the dataset

H_o = All the combinations of noise indicators agree in rating the noise spatial units (in other words, all the noise indicators rank the spatial units from highest to lowest importance).

The application of Kendall's Coefficient of Concordance in SPSS provides the following values for all the combinations with a p-value << 0.001 with a very high agreement in the ranking that can be detected in the total analysis of indicators, W = 0.922. It is considered a high value when W > 0.9.

This allows us to not reject the null hypothesis with 95% of confidence, that is, the hypothesis that there is a total agreement among all the noise indicators in ranking the importance of evaluation points in exposure from highest to the lowest level.

(iv) Test if the spatial vector of the dataset belongs to the same population

H_o = All the noise indicators belong to the same population.

To confirm the claims that have been made, a Friedman test was used to evaluate the differences between indicators, as the dependent variable being measured is ordinal. There was a statistically significant difference in the results provided (by at least two indicators) used, χ2(2) = 8.728, degrees of freedom = 5, and p-value = 0,120 > 0.05 which confirms the acceptance of the null hypothesis. Therefore, it can be claimed that there is not an overall statistically significant difference between the mean ranks of the combination of noise indicators and environmental noise limit values. All the spatial data output of the HSIP 3D-tool belong to the same population.

(v) Clustering analysis results

The hierarchical cluster analysis for dichotomic data follows three steps: 1) calculate the distances for binary data using "Euclidean distance", "pattern difference", "simple matching", and "Jaccard", 2) select the measurement of distance method as an averaged linkage between groups, within groups and centroids clustering linkage, and 3) choose a solution by selecting the right number of (iterations) clusters. Using different approaches confirms the robustness of the conclusions summarized below.

The most different methods among them are those based on L_{den} and the second group formed by L_{night}. The direct result of the quartiles provides the same results from Table S7.

Table S7. Cluster groups. Calculated using the cluster methods: Averaged linkage within groups (and between groups) and the distance measurement, i.e., Jaccard Method. The maximum number of iterations is 10.

Cluster groups	Cluster members per group	Characteristics

Number 1	HSD_CS_58, HSD_58 and Pop_L_{night}_58	3 members based on L_{night}
Number 2	Pop_L_{den}_68, HA_68, HA_CS_68	3 members based on L_{den}

The minimum number of iterations to reach one single group is 25. All these results were confirmed with DTW distance analysis and corroborated by other hierarchical cluster analysis configurations of the raw data.

File S9. Conversion between L_{den} and L_{dia}

The highway case study shows that the traffic and its composition are very similar during the day and evening periods. This particular distribution of traffic allows the translation from L_{den} to L_{day} to be a very simple operation (Figure S11). This AADT shape is not an isolated case and is generalizable (with certain precautions) to other high-density highways in Quito [57].

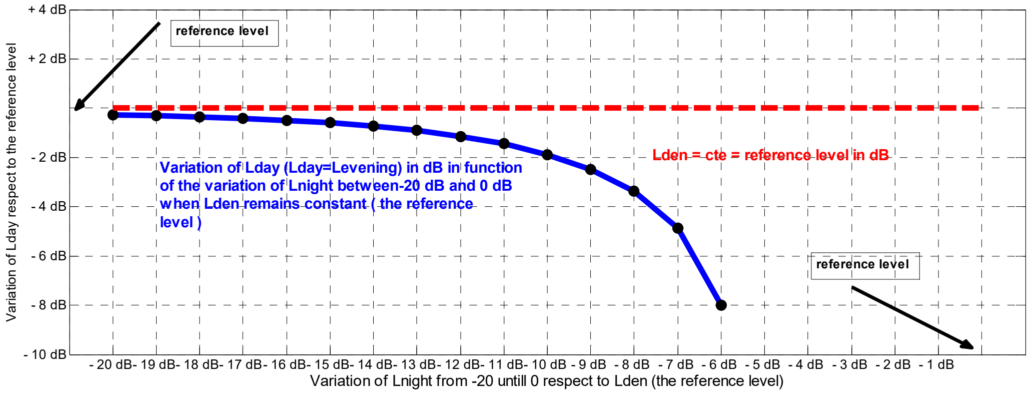

Figure S5 This graph helps to transform L_{den} to L_{day} as a function of the arithmetic difference between L_{den} and L_{night} (in the special case in which L_{day}=$L_{evening}$).

In the case study, $L_{nigth} \approx L_{den} - 10$ dB and *t*he equivalences are defined in Table S8.

Table S8. Equivalence between noise indicators.

Indicator to estimate	The output of noise mapping	The timetable of traffic behavior in vehicles/hour	Indicator equivalences
L_{dia}	L_{den}	07-23h, 5.85 % v/h<br>07-21h, 5.7 % v/h	$L_{dia} \approx L_{den} - 2.2$ *dB*

File S10. Results of the noise maps exposition for L_{night} and L_{noche}

CADNA was used in the development of new noise maps in which the population exposed at L_{noche} were directly calculated (keeping all the parameters of the calculation fixed, except for the reference time periods). The results of the comparisons can be seen in Figures S6 and S7.

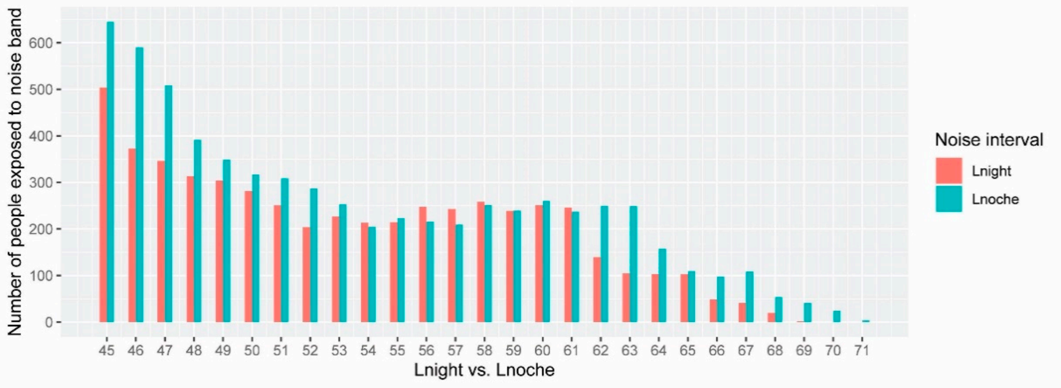

Figure S6. People exposed in the area of study to L_{night} and L_{noche}.

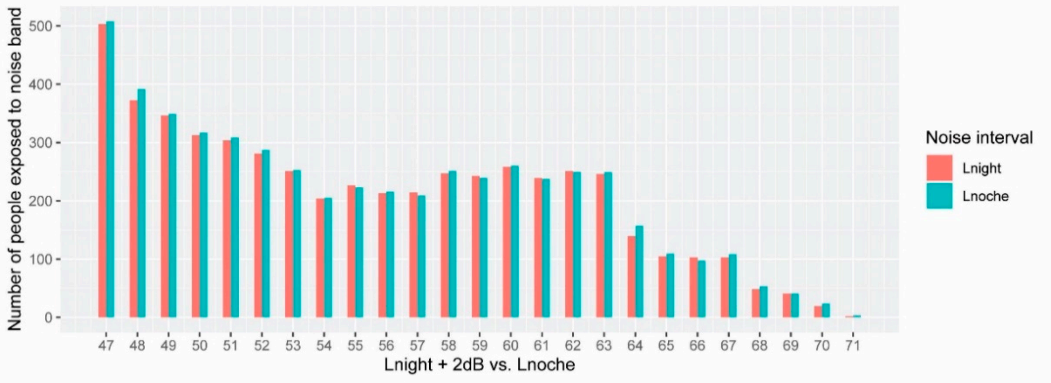

Figure S7 People who were exposed to L_{noche} after the correction of L_{night} + 2 dB (in blue) and the estimation from CADNA of L_{noche} (in red).

According to the results, there is a mismatch between the schedule of daily activities of the inhabitants of Quito and the period marked as L_{noche}. This is shown by traffic data. The legislation has set a time reference to protect the hours of sleep that really should not be taken into account as the sleeping period, at least not in relation to road noise and other indicators of a societal timetable (for example, prime time television).

References

1. German Federal Gazette. Preliminary Calculation Method for Determination of the Number of Persons Exposed to Environmental Noise (Vorläufige Berechnungsmethode Zur Ermittlung Der Belastetenzahlen Durch Umgebungslärm (VBEB)). **2007**, *75*.
2. The European Commission. Commission Directive (EU) 2020/367 of 4 March 2020 Amending Annex III to Directive 2002/49/EC of the European Parliament and of the Council as Regards the Establishment of Assessment Methods for Harmful Effects of Environmental Noise. *Off. J. Eur. Union* **2020**, 132–136.
3. Puyana-Romero, V.; Cueto, J. L.; Gey, R. A 3D GIS Tool for the Detection of Noise Hot-Spots from Major Roads. *Transp. Res. Part D* **2020**, *84*, 102376. https://doi.org/10.1016/j.trd.2020.102376.
4. Probst, W. QCITY - A Concept for Noise Mapping , Ranking , Hot Spot Detection and Action Planning. In *19 th International Congress on Acoustics*; Madrid, Spain, 2007; pp 1–5.
5. Weber, M.; Jabben, J. An Indicator for Area Specific Noise Impact: Gden. In *Internoise 2010*; Lisbon, Portugal, 2010.
6. World Health Organization. European Union. *Environmental Noise Guidelines for the European Region*; 2018.
7. European Commission. *Position Paper on EU Noise Indicators*; 2000.
8. Revelle, W. Psych: Procedures for Personality and Psychological Research. Northwestern University, Evanston, Illinois, USA, 2016.
9. Dalgaard, P. Introductory Statistics with R. In *Statistics and Computing*; Springer, 2008.
10. Marshall, E. The Statistics Tutor's Quick Guide to Commonly Used Statistical Tests.

11. Wei, T.; Simko, V. R Package "Corrplot": Visualization of a Correlation Matrix. 2021.
12. R Core Team. R: A Language and Environment for Statistical Computing. R Foundation for Statistical Computing, Vienna, Austria 2013.
13. Cassisi, C.; Montalto, P.; Aliotta, M. Similarity Measures and Dimensionality Reduction Techniques for Time Series Data Mining. **2012**, No. May 2014. https://doi.org/10.5772/49941.
14. Javed, A.; Suk, B.; Rizzo, D. M. Machine Learning with Applications A Benchmark Study on Time Series Clustering. *Mach. Learn. with Appl.* **2020**, *1* (May), 100001. https://doi.org/10.1016/j.mlwa.2020.100001.
15. Kendall, M. G. *Rank Correlation Methods*, 3rd editio.; Charles Griffin: London, 1962.
16. Hollander, M.; Wolfe, D. A. *Nonparametric Statistical Methods*; John Wiley & Sons, I., Ed.; 1999.
17. Liao, T. W. Clustering of Time Series Data — a Survey. **2005**, *38*, 1857–1874. https://doi.org/10.1016/j.patcog.2005.01.025.
18. Özkoç, E. E. Clustering of Time-Series Data. In *Statistical Machine Learning*; IntechOpen, Ed.
